# The impact of *Tamarix* invasion on the soil physicochemical properties

**DOI:** 10.1038/s41598-022-09797-3

**Published:** 2022-04-06

**Authors:** Tesfay Araya, Asiphe V. Mlahlwa, Mohamed A. M. Abd Elbasit, Solomon W. Newete

**Affiliations:** 1 Department of Soil- and Crop- and Climate Sciences, University of the Free State, PO Box 339, Bloemfontein, 9300 South Africa; 2 Department of Agronomy, University of Fort Hare, Private Bag X1314, Alice, 5700 South Africa; 3 School of Natural and Applied Sciences, Sol Plaatje University, Kimberley, 8301 South Africa; 4 Agricultural Research Council-Natural Resources and Engineering (ARC-NRE), Geo-Information Science Programme, Arcadia, Private Bag X79, Pretoria, 0001 South Africa; 5 School of Animal, Plant and Environmental Sciences, University of the Witwatersrand, Private Bag X3, Johannesburg, 2050 South Africa

**Keywords:** Ecology, Plant sciences, Ecology, Environmental sciences

## Abstract

The exotic *Tamarix* species, *T*. *ramosissima* and *T. chinensis,* were introduced into South Africa in the early 1900s reportedly either for ornamental or soil wind erosion control purposes in the mines. They are, however, currently invading several riparian ecosystems in the country and threatening its biodiversity and proper functioning. The objective of this study was to assess the effects of the exotic *Tamarix* species on the soil physicochemical properties vis-à-vis the indigenous *Tamarix* at the Leeu River in the Western Cape Province, of South Africa where they are purvasive. Three transects were laid from the riverbank towards the outer land, where the exotic followed by the native *Tamarix* species predominantly occurred. Soil was sampled from three points per transect and three soil depths (0–10, 10–20 and 20–30 cm) per point in winter and summer to determine selected soil physicochemical properties. The results showed that total nitrogen (TN), total carbon (TC), Sodium (Na), Potassium (K) and Magnesium (Mg) concentrations under the native and exotic *Tamarix* species were significantly higher than those in the open land without *Tamarix *species. The salinity under the native and exotic *Tamarix* species was greater (P < 0.05) in the topsoils (0–10 cm) than in the deeper soils (20–30 cm) with 5.05 mS cm^−1^ and 4.73 mS cm^−1^, respectively. Soil electrical conductivity (EC) was higher (P < 0.05) during the winter season under the exotic *Tamarix* species (5.05 mS cm^−1^) followed by the native species (4.73 mS cm^−1^) and it was the lowest in the control (0.16 mS cm^−1^) at 0–10 cm soil depth. Similarly, sodium and sodium absorption ratios (SAR) under the native and exotic *Tamarix* species were significantly greater than those in the control. The highest levels (P < 0.05) of TC were recorded at the topsoil (0–10 cm soil depth) under the exotic *Tamarix* species (1.17%), followed by the native *Tamarix* (1.07%) with the control recording the lowest (0.53%). There were no significant differences (P < 0.05) in K, TC, TN and SOC concentrations at lower soil depths (20–30 cm). The soil texture was significantly affected by the *Tamarix* species. The soil bulk density was lower under the exotic *Tamarix* followed by native *Tamarix* species than the control soils. The soil volumetric water content was higher under the exotic *Tamarix* species compared to the control. This study concludes that the invasion of the exotic and native *Tamarix* species altered the soil properties underneath and created conducive soil conditions for their predominance.

## Introduction

The widespread of invasive species and their economic impact is well documented^1–3^. They are considered as the second major global threat to the ecosystem and biodiversity after direct habitat destruction^4,5^. The genus *Tamarix,* with over 54 known species, is one of the four genera in the family of Tamaricaceae, native to the Euro-Asia regions and some parts of Africa^6,7^. Many of its species, however, have already spread outside their natural habitat across the world, imported as shade, erosion control, and ornamental plants^7–9^. For instance, *Tamarix* was first imported into the United States as an ornamental plant in the early 1800s and later as a windbreak and land stabilization plant to reduce wind and water erosion^10^. In South Africa, the exotic *Tamarix* species (*Tamarix ramosissima* and *T. chinensis*) are believed to have been introduced in the early 1900s as an ornamental or phytoremediation plant^7,9^. Alien invasive plants in South Africa not only alter soil biological, physical and chemical properties but also lead to a significant decline in freshwater and wetland ecosystems^11^. There are some studies supporting that invasive plants are capable of altering soil physicochemical properties, such as the nitrogen cycle and other various elements^12–14^, pH^15^, soil organic matter (SOM) and soil aggregation^16^. However, no study is conducted on the impacts of different *Tamarix* species on the soil physicochemical properties in the context of Southern Africa soils where these invasive alien species are widely distributed.

The spread of invasive *Tamarix* species has been linked to several environmental challenges, including alteration in river morphology, elevated soil salinity levels, replacement of native vegetation and increase in fire frequencies^17^. Soil salinization causes a major threat to biodiversity, and this could have a detrimental effect on the economy in countries such as South Africa, where the vast biodiversity hostspots play important role in the tourism sector of the economy^18^. The two exotic *Tamarix* species in South Africa, have been declared as category 1b invasive weeds by the National Environmental Management: Biodiversity Act 2014 (NEM: BA)^9^ and their ability to hybridize with the native *T*. *usneoides*^19^ poses a potential ecological threat of diluting the parental genetic pool of the native *Tamarix* in the country. Species categorized under category 1b are known as established invasive species, which must be removed immediately.

The legacy of invasive alien plants on the physicochemical soil properties is often complicated, and restoration takes time. The impacts of high salinity levels in soils previously invaded by halophytes may persist for a longer period even after the invasion is brought under control^20,21^, making the clearance of alien invasive species and restoration of the ecosystem complicated^22^. Even after implementing management programmes such as control or eradication, the footprints left by invasive *Tamarix* species could lead to secondary invasion^23,24^. Since the soil will be left highly susceptible to other invasions, the bare soils after clearance of invaders become unsuitable for restoration and re-establishment of indigenous species^25^. Thus, it is essential to understand their legacy after managing soil properties to rehabilitate cleared areas from invasive species successfully. Although little is known about the actual impact of *Tamarix* on soil properties in South Africa, many studies have focused on their ecological and water resource impact. Understanding the changes in soil properties due to *Tamarix* invasion is a prerequisite for successful soil rehabilitation. This study therefore, investigated the impact of the native and exotic *Tamarix* species on soil physicochemical properties in the Leeu River, Western Cape Province, and provides an insight into the soil alterations caused by *Tamarix* species.

## Materials and method

### Site description

The study was conducted in the summer of 2018 and winter of 2019 at Leeu River (32° 46′ 04″ S, 021° 58′ 46″ E) located near the town of Leeu Gamka on the N1 road about 100 km from the town of Prince Albert in the Western Cape Province. The soil textural class under the study is dominantly sandy loam texture. The Leeu Gamka area with little rainfall all year round receives approximately 146 mm of rain annually. The temperature ranges from 14 to 30 °C in summer (November to March) and 2–23 °C in winter (May to August).

### Soil sampling

**Figure 1:**
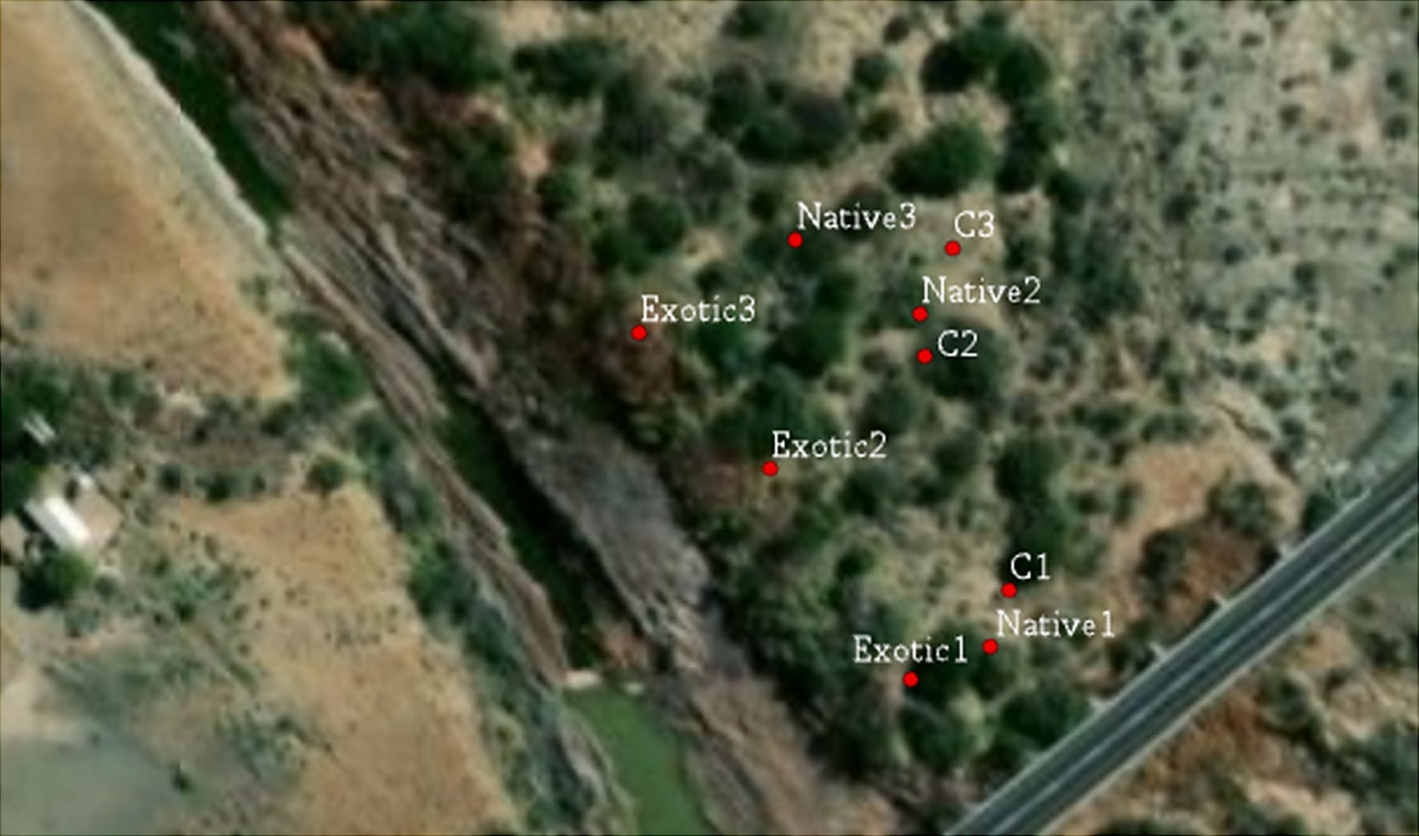
Soil sampling points along transect in Leeu River in the Western Cape Province. Exotic (1, 2 and 3) is exotic *Tamarix* species, native (1, 2 and 3) is native *Tamarix* species and C (1, 2 and 3) is control (*Tamarix* free land).

Composite soil samples were collected from three transects (30 m apart) laid across the Leeu River from the water point towards the *Tamarix* species at the river bank at three points per transect at an interval of 20 m and three soil depths (0–10 cm, 10–20 cm and 20–30 cm) per point (Fig. 1) to determine the effect of *Tamarix* species on selected soil physicochemical properties. The sampling was conducted in summer and winter season. The study considered the topsoil at three depths because most of the changes in the soil chemical and physical properties could also be associated with the impacts of the *Tamarix* leaves directly deposited on the soil surface. The soil samples were collected from soils under the native *T. usneoides* and the exotic *T*. *ramosissima* and *T*. *chinensis* and the control soils (*Tamarix* free soils) further away from the *Tamarix* species. Transects were laid from the riverbank's edge towards the outer land (Fig. 1). The first soil sampling was taken 3 m away from the riverbank to exclude the direct impact of the flood plain soils. The exotic *Tamarix* species predominantly occurred along the riverbank, while the native *Tamarix* occurred further away from the edge of the riverbank following the exotic *Tamarix* species.

### Soil chemical analysis

Composite soil samples were passed through a 2 mm sieve to remove plant debris, gravel or any foreign object in the sample before the soil chemical analysis. The soil pH readings were taken using a pH meter (XS Instruments, Italy) in a 1:2.5 soil: water suspension as outlined by AgriLASA^26^. Soil electrical conductivity (EC) was determined using a conductivity meter (XS Instruments, Italy) on the same suspension used for pH reading^27^. Air-dried and grounded soil samples were used to determine total carbon (C) and nitrogen (N) according to the dry combustion method using a LECO auto analyzer^28^. The modified Walkley–Black method was used to analyze soil organic carbon (SOC) using the procedures described in^26^.

Sodium (Na^+^), potassium (K^+^), calcium (Ca^2+^), and magnesium (Mg^2+^) cations were extracted using ammonium acetate buffered at pH 7 using the procedures outlined in AgriLASA^26^, and their concentrations were determined using an Inductively Coupled Plasma–Atomic Emission Spectrograph (ICP–OES) (Varian Inc. The Netherlands). The SAR was calculated using Eq. (1)^29^.

1 $$ {\text{SAR}}\left( {{\text{Cmol}}\left( + \right)/{\text{kg}}} \right) = \frac{{{\text{N}}a^{ + } }}{{\sqrt {\frac{{Ca^{2 + } + Mg^{2 + } }}{2}} }} $$

### Soil physical properties

Undisturbed soil samples were collected to determine the bulk density (ρ_b_) and calculated using Eq. (2)

2 $${\rho }_{b}=\frac{{M}_{s}}{{V}_{t}}$$

where M_*s*_ is the mass of dry soil and V_*t*_ is the volume of 100 cm^3^ soil core cutter^30^.

The soil volumetric water content was determined using the gravimetric method at 0–5 cm soil depth^31^. The soil gravimetric water content and volumetric water content were calculated using Eqs. (3) and (4), respectively

3 $${\uptheta }_{\mathrm{g}}=\frac{{M}_{w}}{{\rho }_{s}}$$

where M_*w*_ is the mass of water and M_*s*_ is the mass of dry soil

4 $${\theta }_{v}={\theta }_{g}\frac{{\rho }_{b}}{{\rho }_{w}}$$

where θ_g_ is the gravimetric water content, *ρb* is the soil bulk density and *ρw* is density of water (1 g cm^−3^).

Soil particle size was analyzed using the hydrometer method as described by Okalebo et al.^27^. The soil texture was determined from sand, silt and clay percentages using soil textural triangle.

### Data analysis

Data analysis was performed using JMP statistical package version 14.0 (SAS Institute, Inc., Cary, NC, USA). Factorial Analysis of variance (ANOVA) was performed on all variables as multiple categorical independent variables were present and followed by a post hoc test using the Turkey (HSD) test when the ANOVA results are significant at 95% level of confidence (P < 0.05).

## Results

### Effect of *Tamarix* species on soil pH and salt levels

**Figure 2:**
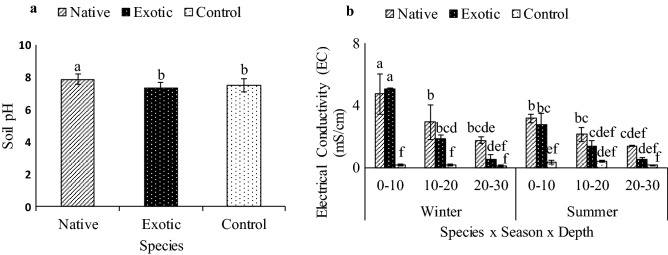
Effects of *Tamarix* species (native and exotic) on (**a**) soil pH and (**b**) electrical conductivity in Leeu River at three soil depths (0–10, 10–20 and 20–30 cm), in two seasons (winter and summer). Means followed by different letters are significantly different from each other (Turkey’s test HSD). Bars indicate standard deviation from the means.

The soils were generally alkaline, with pH values between 7.3 and 7.9. The native *Tamarix* species had a significantly higher soil pH (7.9) compared to the soils in the control (soils with no *Tamarix* species) (pH of 7.5) and to those under the exotic *Tamarix* species (pH 7.3) (Fig. 2a). The soil EC was significantly higher in both the native and exotic *Tamarix* species compared to the control (P < 0.05). The highest soil EC was observed at 0–10 cm soil depth during the winter season under the exotic (5.05 mS cm^−1^) followed by the native (4.73 mS cm^−1^) *Tamarix* species (Fig. 2b). The control had the lowest (0.16 mS cm^−1^) soil EC record. In summer, however, the soil EC at the topsoil (0–10 cm depth) decreased by 45% and 38%, respectively compared to those recorded in winter. A general trend of decrease in soil EC was obsereved with the increase of soil depth in all treatments (under the *Tamarix* and control soils) regardless of the season.

### Effects of *Tamarix* species on sodium and sodium adsorption ratio

**Figure 3:**
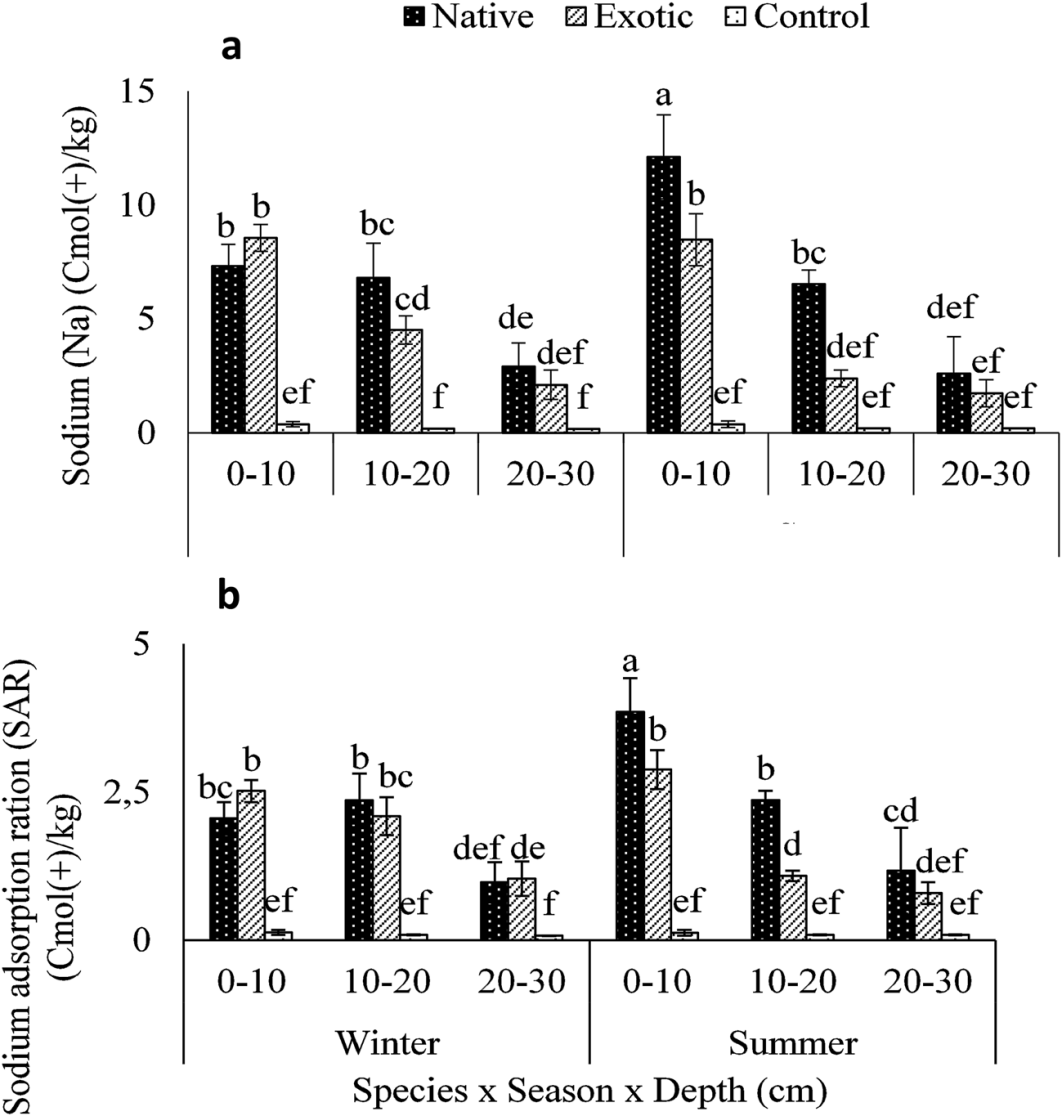
Three way interaction (Species × Season × Depth) *Tamarix* species (native and exotic) on (**a**) sodium and (**b**) sodium adsorption ratio at three soil depths (0–10, 10–20 and 20–30 cm) in two seasons (winter and summer) in Leeu River. Means followed by different letters are significantly different from each other, with separation of means performed using Turkey’s test HSD. Bars indicate standard deviation.

The Sodium and SAR under native and exotic *Tamarix* species were higher than those observed under the control conditions. The highest Na and SAR levels were recorded at 0–10 cm soil depth and decreased thereafter with an increase in soil depth. The Na levels at the top 0–10 cm soils under all treatments including the controls were recorded in the range of 0.37–8.5 cmol (+) kg^−1^ in winter and 0.37–12.1 cmol (+) kg^−1^ (Fig. 3a) in summer. The control soil had the lowest record of Na levelsin both winter and summer seasons. The soils under the exotic *Tamarix* species had the highest Na in winter, while the soil under native *Tamarix* species had the highest Na levels in summer. The SAR at 0–10 cm soil depth increased by 23% under the exotic *Tamarix* species in winter compared to the native *Tamarix* species, while it decreased by 34% in summer (Fig. 3b). Na and SAR levels observed under the exotic *Tamarix* species and control showed no significance with the season changes. However, both showed significant differences at 0–10 cm soil depth under the native *Tamarix* species in summer.

### Effects of *Tamarix* species on extractable cations

**Figure 4:**
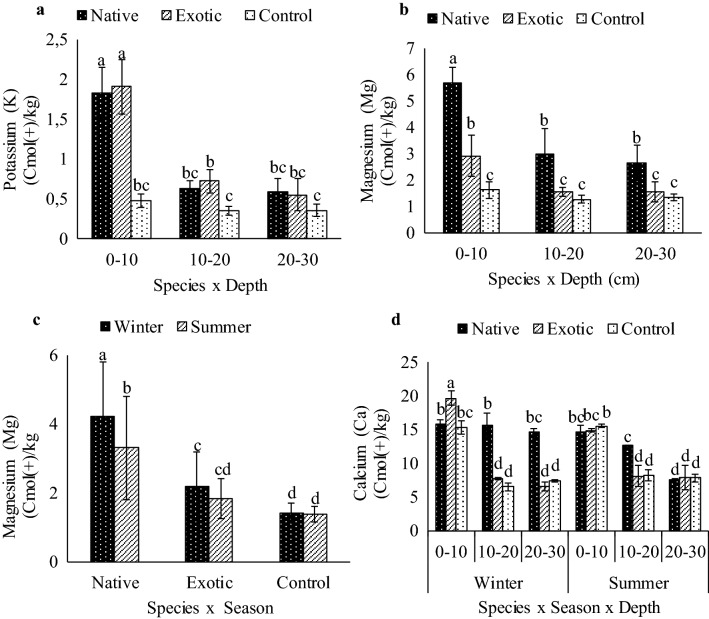
Influence of *Tamarix* species (exotic and native) on extractable cations (**a**) two way interaction (species × depth) on potassium, (**b**) two way interaction (species × depth) on magnesium, (**c**) two way interaction (species × season) on magnesium and (**d**) three way interaction on calcuim (Species × Season × Depth) at three soil depths (0–10, 10–20 and 20–30 cm) in two seasons (winter and summer). Means followed by different letters are significantly different from each other, with separation of means performed using Turkey’s test HSD. Bars indicate standard deviation.

The soil K content decreased with an increase in soil depth, and the highest K was observed at the topsoil (0–10 cm depth) with thereafter showing no significant difference between the 10–20 cm and 20–30 cm soil depths (Fig. 4a). Potassium at the topsoil (0–10 cm depth) increased significantly under both the exotic and native *Tamarix* species compared to the control. The highest K was observed under the exotic (1.91 cmol (+) kg^−1^)) followed by the native (1.80 cmol(+) kg^−1^)) *Tamarix* species, while the lowest (0.48 cmol(+) kg^−1^)) was recorded under the control soils. The the K levels were 1.35 and 1.43 cmol(+) kg^−1^ higher under the native and exotic *Tamarix* species, respectively than those in the control soils.

Similarly, the soil Mg content decreased with an increase in soil depth (Fig. 4b). The highest Mg (4.2 cmol(+) kg^−1^) was recorded under native *Tamarix* species at 0–10 cm soil depth, which was significantly higher than those under exotic *Tamarix* species (2.19 cmol(+) kg^−1^)) and the control soils including in the rest of the soil depth profiles (10–20 and 20–30 cm). There was no significant difference in Mg levels under the soils between the exotic *Tamarix* and the controls (1.42 cmol(+) kg^−1^)) observed in winter. However, the Mg levels under the exotic, native and control soils decreased by 18%, 21% and 2%, respectively, in summer compared to those in winter (Fig. 4c).

The highest Ca was found at 0–10 cm soil under the exotic *Tamarix* species (20 cmol(+) kg^−1^) followed by the native (16 cmol(+) kg^−1^) with the lowest under the control soils (15 cmol(+) kg^−1^) (Fig. 4d). The Ca soil concentration decreased with the increase in soil depth, and the lowest was observed at 20–30 cm soil depth in both seasons. The highest Ca levels were observed at 0–10 cm soil depth under the exotic *Tamarix* species. Ca levels at 0–10 cm soil depth during the winter season showed no significant difference between the soils under the native *Tamarix* species and the control. However, there was no significant difference in Ca concentrations in soils under the control and *Tamarix* species in summer.

### Effects of *Tamarix* species on total carbon, total nitrogen and soil organic carbon

**Figure 5:**
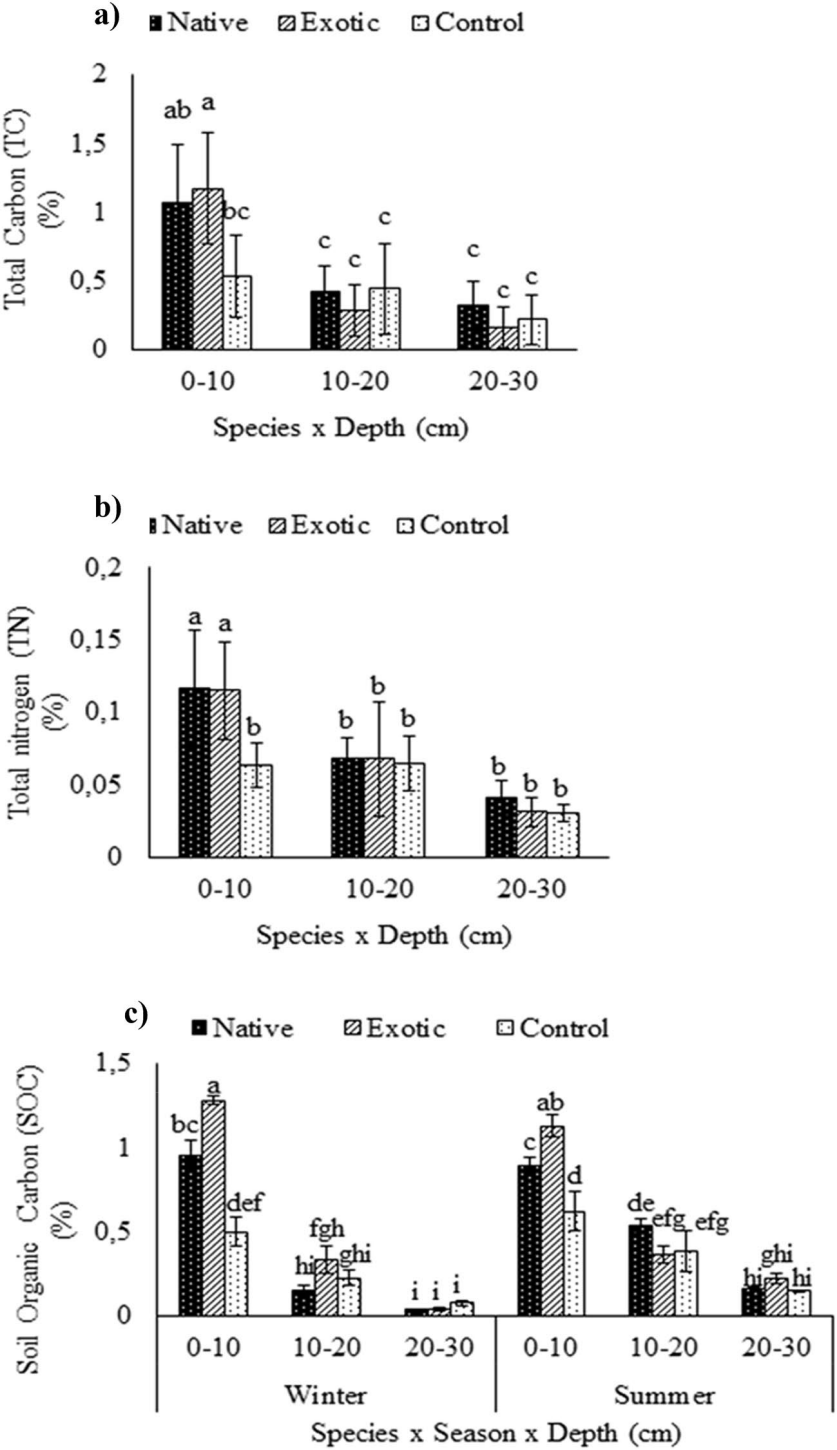
(**a**) The interactive effects of species × soil depth on total carbon, (**b**) Total nitrogen and (**c**) The interactive effects of species × season × soil depth on soil organic carbon under native *Tamarix*, extotic *Tamarix* and the control at three soil depths (0–10, 10–20, 20–30 cm) in two season at Leeu River, Western Cape. Means followed by different letters are significantly different from each other, with separation of means performed using Turkey’s test HSD. Bars indicate standard deviation.

The TC, TN, and SOC decreased with increased soil depth. The highest levels of TC were recorded at the topsoil (0–10 cm soil depth) under the exotic *Tamarix* species (1.2%), followed by the native *Tamarix* (1.1%). It was the lowest (0.53%) under the control soils (Fig. 5a). The TC significantly decreased by 70%, 86%, and 59% from 0 to 10 cm, 10–20 cm and 20–30 cm under the native *Tamarix*, exotic *Tamarix* and the control soils, respectively (Fig. 5a). There were no significant differences in TC, TN and SOC concentrations at lower soil depths (20–30 cm) observed between the control and the two *Tamarix* species. The SOC increased by 0.46% and 0.81% under the native and exotic *Tamarix* species, respectively, compared to the control soils in winter and increased by 0.27% and 0.51% in summer (Fig. 5c). However, there was no significant differences in SOC between the two seasons at 0–10 cm and 20–30 cm soil depth.

### Effects of *Tamarix* species on soil texture, bulk density and soil water content

The soil texture was significantly affected by the *Tamarix* species. The soil texture under the control soil was sandy loam soils with an average of 20% (silt) and 6% (clay) content. The soil texture under the native *Tamarix* was silt loam, while the soil under the exotic *Tamarix* was loam texture and sandy loam texture. The clay content under the exotic (15.8%) was less than the native (11.3%) *Tamarix* species, while the silt under the native (57.4%) *Tamarix* was significantly higher compared to the soils under the exotic *Tamarix* (43.5%) and control (20.7%) (Table 1).

**Table 1:** Soil textural composition percentage and soil textural class observed under different *Tamarix* species and the control (open-land).

Species	Sand%	Silt %	Clay%	Textural class
Native	31.3	57.4	11.3	Silt loam
Exotic	40.7	43.5	15.8	Loam
Control	72.8	20.7	6.5	Sandy loam

**Figure 6:**
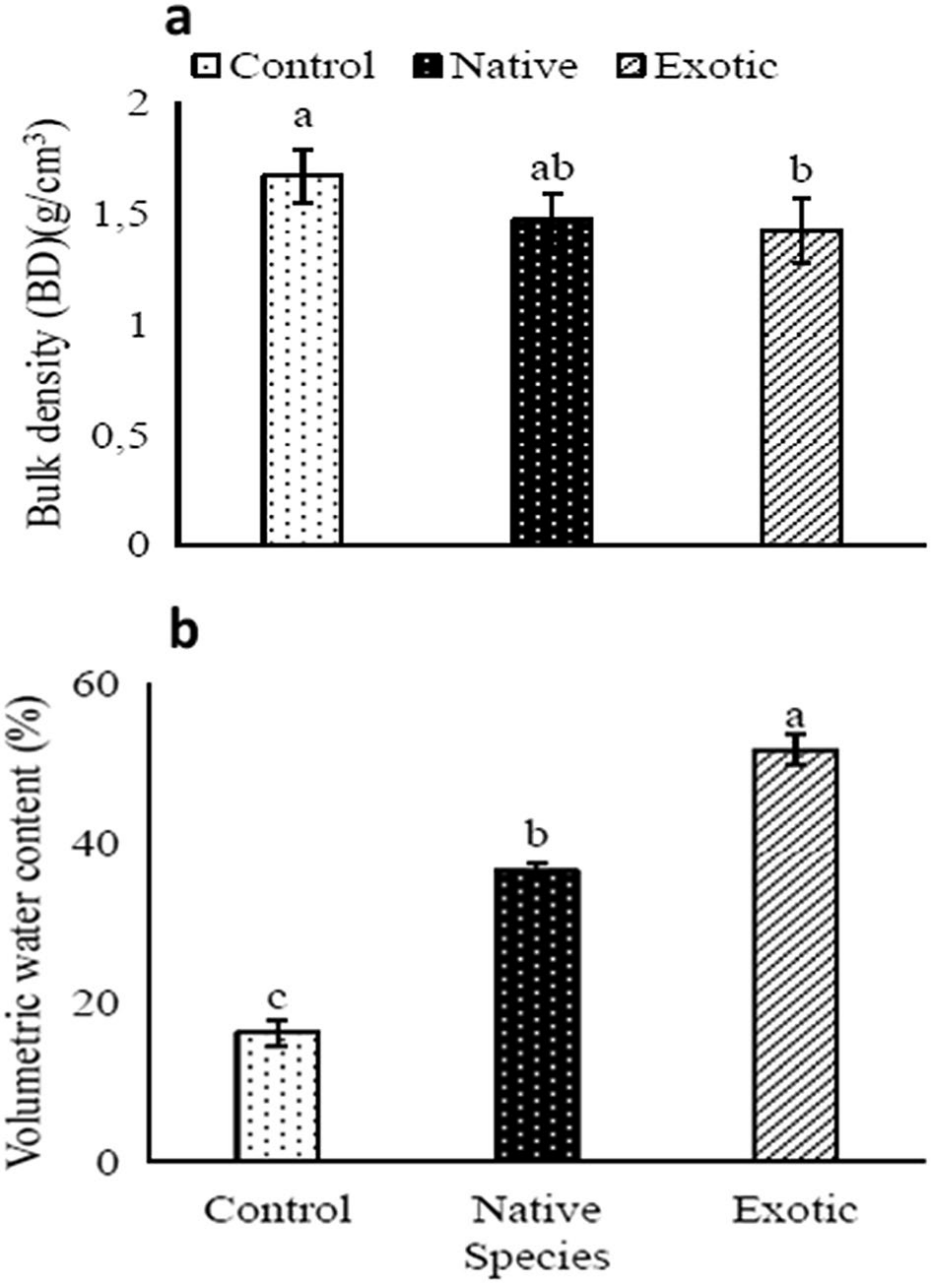
The effects of two different *Tamarix* species on soil bulk density and volumetric water content compared to the controls: (**a**) Soil bulk density and (**b**) soil volumetric water content. Means followed by different letters are significantly different from each other (Turkey’s test HSD). Bars indicate standard deviation.

The soil bulk density under the exotic *Tamarix* species decreased significantly by 18% compared to the control soils. However, there was no significant difference between the native *Tamarix* and control soils. Similarly, the soil bulk density between the soils under the exotic and native *Tamarix* was not significantly different (Fig. 6a).

The soil volumetric water content was significantly higher in soils under the exotic and native *Tamarix* species by 36% and 20%, respectively, compared to the control (Fig. 6b) with native *Tamarix* species.

## Discussion

### Effect of *Tamarix* species on soil pH and soil salt levels

The soil pH increased with distance further away from the exotic *Tamarix* species towards the native species (Fig. 2a). The native *T. usneoides* has little leaf litter under its canopy compared to the exotic *Tamarix* species since the native is an evergreen species^32^. The exotic *Tamarix* trees had an enormous amount of foliar litter under their canopy as opposed to the isolated individual native *Tamarix* trees. The soil pH under the native *Tamarix* species was significantly higher than those observed in soils under the exotic *Tamarix* species. The decrease in soil pH under the exotic *Tamarix* could be explained by the addition of organic acids from decaying leaf litter (Fig. 2a)^33^ and the release of carbon dioxide (CO_2_) during microbial decomposition, which produces carbonic acid that promotes the release of hydrogen ions (H^+^). On the other hand, the increase in soil pH under the native *Tamarix* can be associated with relatively low leaching of base cations such as Na, Mg and Ca^34^ that have accumulated through the leaf litter.

The exotic and native *Tamarix* species showed a significant increase in EC compared to the control soils. This was in line with Liu et al.^35^, who reported an increase in EC in soils under *Tamarix* species. It is generally known that soluble salts are naturally present in semi-arid and arid regions and tend to accumulate on the soil surface due to high evaporation and low rainfall for leaching surface salt concentrations into deeper soil horizons. However, *Tamarix* species extracted soil salts from the lower horizon and deposited them on the soil surface through leaf litter deposition^36–38^, resulting in higher salinity levels under *Tamarix* canopies compared to the controls. *Tamarix* species have a characteristic adaptation strategies to extract chemical elements from deeper soil profiles, which are eventually excreted through their foliar salt glands^36,39,40^. These salt depositions significantly alter soil chemistry, microbial activities and species diversity of the habitat.

### Effects of *Tamarix* species on soil exchangeable cations

An increase of exchangeable cations was observed under the exotic and native *Tamarix* species compared to the control. The cation (K^+^, Mg^2+^, and Na^+^) levels were significantly higher under the native and exotic *Tamarix* species than in the control soils at the top 0–10 cm depth, except for Ca^2+^, which was not significantly different between the control and native *Tamarix* species in winter. This indicates that *Tamarix* species are capable of increasing cation levels on the soil surface through salt excretion and litter decomposition^33,36,41–43^ and altered soil chemical properties^44,45^. Exchangeable cations (Ca^2+^, K^+^, Mg^2+^, Na^+^) deposition from *Tamarix* species decreased with increase in soil depth, resulting in higher concentrations on the topsoil, which is in agreement with the findings of other similar studies^46–48^.

### Effects of *Tamarix* species on sodium adsorption ratio

The SAR levels on the topsoil (0–10 cm) were significantly lower under the control in both winter and summer seasons compared to the native and exotic *Tamarix* species. This shows an increase in salt (Na^+^) enrichment under *Tamarix* species. However, our findings are different from those reported by Zhang et al.^49^, who reported that Na^+^ to be the most dominant ion under *T. ramossissma*. However, they reported that the soil in their study was a Na^+^ dominated soils as opposed to the soil in the current study which was Ca^2+^ dominated soil. SAR is primarily influenced by the selective absorption of salt ions, which also depends on the available ions in the soil. The SAR in winter was 2.05 cmol(+) kg^−1^ and 2.5 cmol(+) kg^−1^ at the top 0–10 cm soil depth under the native and exotic *Tamarix,* respectively. In contrast, those of the control soils were 0.132 cmol(+) kg^−1^ suggesting that *Tamarix* species can promote Na^+^ hazards.

### Effects of *Tamarix* species on soil organic carbon, total nitrogen and total carbon

The SOC, TN and TC were significantly higher in the topsoil (0–10 cm) and decreased with increasing soil depth. TN was not significantly different between the 10–20 cm and 20–30 cm soil depths (Fig. 5b). The invasion of *Tamarix* species increased in soil nutrient levels under their canopy^43,44,50^. The increase in SOC, TN and TC levels under the native and exotic *Tamarix*, when compared to the control at the topsoil, was mainly due to the leaf litter deposition and foliar guttation as well as accumulation of SOC under the *Tamarix* species.

### Effect of *Tamarix* species on soil texture, bulk density and soil water content

The soil texture under the exotic *Tamraix* species was loam. The soil under native *Tamarix* species was silty loam in texture while the sandy texture in soils under the control demonstrated the impacts of *Tamarix* invasion. Unlike the effect of the seasons (winter and summer), the difference in *Tamarix* species had a significant effect on the soil bulk density. The lower soil bulk density under *Tamarix* species compared to the control was mainly due to high soil organic matter and high silt and clay content^51–53^. Thus, our results revealed that as soil organic matter and clay content increased under *Tamarix* species soil bulk density decreased.

The soil volumetric water content under the *Tamarix* species differed significantly from that in the control. The volumetric water content was in the order of control < native < exotic *Tamarix* species.

## Conclusion

The invasion of *Tamarix* species had a significant impact on the underneath soil physicochemical properties. The soil salinity, Na^+^, Mg^2+^, Ca^2+^, K^+^, TC, TN and SOC were greater at the topsoil (0–10 cm) than in the deeper soils (10–20 cm and 20–30 cm) under the *Tamarix* canopes as well as the controls. These results suggest that the effects brought by *Tamarix* species were primarily limited to the topsoil due to the foliar guttation, leaf litter deposition and decomposition. The magnitude of the cations found in the soils under both *Tamarix* species were in the order of Ca^2+^ > Na^+^ > Mg^2+^  > K^+^ and under the control were in the order Ca^2+^ > Mg^2+^  > K^+^  > Na^+^, which demonstrates that the presence of more Na^+^ in the soils under *Tamarix* species compared to the control. The soils in the study site under the control have generally higher Ca^2+^ and Mg^2+^ compared to the soils underneath the *Tamarix* species. The results revealed that *Tamarix* species could alter the soil properties making it conducive for their growth. There was no significant difference in soil EC under the exotic *Tamarix* compared to the native *Tamarix* species. However, the SOC was significantly higher in soils under the exotic *Tamarix* species than the soils under the native *Tamarix* species because of the higher leaf litter biomass deposition and decomposition under the exotic *Tamarix* species*.* Similarly, there was higher Na^+^ in summer under exotic as compared to the native *Tamarix* species. The soil texture was significantly affected by the *Tamarix* species. Soil bulk density was lower under the *Tamarix* species than the control. Soil volumetric water content was higher under *Tamarix* species than in the control. The invasion of exotic and native *Tamarix* species have changed the soil physical and chemical properties favouring the predominance of the invasive *Tamarix* species.
